# Disorder predispositions and protections of Labrador Retrievers in the UK

**DOI:** 10.1038/s41598-021-93379-2

**Published:** 2021-07-14

**Authors:** Camilla Pegram, Charlotte Woolley, Dave C. Brodbelt, David B. Church, Dan G. O’Neill

**Affiliations:** 1 Pathobiology and Population Sciences, The Royal Veterinary College, Hawkshead Lane, North Mymms, Hatfield, AL9 7TA Herts UK; 2 The Roslin Institute and the Royal (Dick), School of Veterinary Studies, The University of Edinburgh, Easter Bush Campus, Midlothian, EH25 9RG UK; 3 Clinical Sciences and Services, The Royal Veterinary College, Hawkshead Lane, North Mymms, Hatfield, AL9 7TA Herts UK

**Keywords:** Diseases, Risk factors

## Abstract

The Labrador Retriever is one of the most popular dog breeds worldwide, therefore it is important to have reliable evidence on the general health issues of the breed. Using anonymised veterinary clinical data from the VetCompass Programme, this study aimed to explore the relative risk to common disorders in the Labrador Retriever. The clinical records of a random sample of dogs were reviewed to extract the most definitive diagnoses for all disorders recorded during 2016. A list of disorders was generated, including the 30 most common disorders in Labrador Retrievers and the 30 most common disorders in non-Labrador Retrievers. Multivariable logistic regression was used to report the odds of each of these disorders in 1462 (6.6%) Labrador Retrievers compared with 20,786 (93.4%) non-Labrador Retrievers. At a specific-level of diagnostic precision, after accounting for confounding, Labrador Retrievers had significantly increased odds of 12/35 (34.3%) disorders compared to non-Labrador Retrievers; osteoarthritis (OR 2.83) had the highest odds. Conversely, Labrador Retrievers had reduced odds of 7/35 (20.0%) disorders; patellar luxation (OR 0.18) had the lowest odds. This study provides useful information about breed-specific disorder predispositions and protections, which future research could evaluate further to produce definitive guidance for Labrador Retriever breeders and owners.

## Introduction

Dogs have been domesticated for over 10,000 years, although the exact timeline and location of domestication is still debated^1–4^. During domestication, selective breeding has progressively exaggerated various characteristics and phenotypes of specific subsets of dogs to enhance their usefulness and desirability to humans and has ultimately led to the development of distinct breeds within the canine species^5^. There are now over 350 recognised dog breeds worldwide, which generally show reducing genetic diversity within each breed over time^6^. The narrowing of gene pools through inbreeding has led to concerns about increasing susceptibility to inherited conditions and therefore reducing health overall in purebred dogs. However, the availability of reliable evidence on disorder predispositions and protections within many breeds is limited^7–9^.

The Labrador Retriever is one of the most popular dog breeds worldwide and is lauded for its gentle and social nature, trainability and low aggression^10,11^. It has been the most commonly registered Kennel Club (KC) breed in the UK for some decades; although briefly overtaken in 2018 by the French Bulldog, it soon regained first place in 2019^12^. The breed originated from Newfoundland, Canada, where they were selected by fishermen to retrieve nets and pull carts. Following introduction into the UK in the mid-eighteenth century, the breed gained popularity for use as gundogs and was first recognised by the KC in 1903^13^.

Labrador Retrievers have been regarded historically as a relatively healthy breed^14^. Among the KC-registered subset of dogs in the UK, the median longevity of Labrador Retrievers was reported to be 12.3 years, approximately 1 year longer than the median across all KC-recognised breeds of 11.3 years^15^ However, in recent years, reports on a growing number of inherited diseases have challenged this “healthy” image of the breed^16–26^. Researchers at University of California Davis Veterinary Genetics Laboratory (UCD VGL) report that, while genetic diversity in Labrador Retrievers in the US is reasonable in comparison to other breeds, some bloodlines are more inbred than others, and they used this background to explain why Labrador Retrievers are predisposed to several disorders^14^.

Using anonymised clinical data from primary-care veterinary practices in 2013, the most commonly reported disorders of Labrador Retrievers in the UK were otitis externa, obesity and osteoarthritis^27^. Compared to previous reports that were based on the same data resource, that study suggested that Labrador Retrievers had increased prevalence of otitis externa, lipoma, gastroenteritis and skin laceration at a precise-level of diagnosis and enteropathic, aural, neoplastic and urinary disorders, traumatic injury and obesity at a grouped-level of diagnosis in comparison to Border Collies, Yorkshire Terriers, German Shepherd Dogs, Cocker Spaniels, Jack Russell Terriers, Staffordshire Bull Terriers and crossbreeds^28^. Other studies have reported high prevalence of obesity^17,29,30^, osteoarthritis^31^ and lipoma^27,32,33^ and high incidence of gastrointestinal signs^34^ in Labrador Retrievers. However, prevalence comparisons often fail to compare results against other breeds within the same study or to account for major confounding effects such as age, and therefore offer weak evidence of true relative disorder risk (predisposition or protection)^8^.

Disorder predisposition or protection within breeds can be inferred when there is a respective increased or decreased risk of a condition in a particular breed in comparison with some other relevant comparator group of dogs^8^. Predispositions to 67 disorders are reported for Labrador Retrievers^8^ and the breed also was found to be at a greater risk of diseases of the extremities relative to mixed breed dogs^35^. A large survey of owners of pedigree dogs reported that Labrador Retrievers had a significantly higher prevalence of arthritis, osteoarthritis, elbow and hip dysplasia, lipoma, osteochondritis dissecans and skin cancer than 40 other KC-registered breeds^36^. However, this study also reported that Labrador Retrievers had significantly lower prevalence (i.e. suggestion of protection) of 18 disorders, including anal gland blockages, blocked tear ducts, colitis, corneal ulcers and cryptorchidism. Other studies have reported decreased risk in Labrador Retrievers for benign prostatic hyperplasia^37^, heart murmurs and degenerative mitral valve disease^38^ and fatal acute pancreatitis^39^. However, to date, studies have not reported predispositions and protections in Labrador Retrievers using one dataset, which would avoid compounding biases.

There is some evidence that targeted breeding strategies over recent decades have been associated with increased longevity^40^ and decreased prevalence of hip and elbow dysplasia^41,42^ in Labrador Retrievers. However, a deeper understanding of predispositions and protections among common disorders in Labrador Retrievers offers the potential to teach us as much about how to breed healthier dogs by breeding towards low disorder risk rather than just trying to breed away from high disorder risk as is the current preference in many dog breeding strategies^9,43^. Using anonymised veterinary clinical data on dogs under primary veterinary care in the UK during 2016 from the VetCompass Programme^44^, this study aimed to compare the odds of diagnosis in Labrador Retrievers compared to non-Labrador Retrievers for each disorder in a combined list of the most common disorders in Labrador Retrievers and non-Labrador Retrievers, after accounting for major confounding demographic variables. Demographic data, such as breed, sex and neuter status, is routinely collected in primary-care databases, and therefore large quantities of data can be collected and used to robustly address the demographic risk factors for disorders. However, retrospective primary-care data are less optimal for gathering information on more nuanced risk factors, such as dog behaviour and owner lifestyle, including diet and exercise, and these were therefore not addressed in this study^45^. This new evidence on predispositions and protections could support future research and contribute to better health guidance for Labrador Retriever breeders and owners. In addition, the findings could assist current Labrador Retriever owners to recognise common problems in their dogs, which may lead to earlier veterinary visits, quicker diagnosis and thus better prognosis.

## Results

### Demography

The study included a random sample of 22,333 dogs under veterinary care during 2016 in the UK from a population of 905,544. Breed information was missing for 85 (0.4%) dogs, which were excluded from further analysis, leaving 22,248 dogs (99.6%) in the analysis. There were 1462 (6.6%) Labrador Retrievers, which was the most common purebred breed. The most common breeds amongst non-Labrador Retrievers (20,786; 93.4%) included 1304 (5.9% of the 22,248 dogs) Staffordshire Bull Terriers, 1168 (5.2%) Jack Russell Terriers, 793 (3.6%) Shih-tzus, 771 (3.5%) Cocker Spaniels, along with 5981 (26.9%) crossbreeds. Data completeness for these 22,248 dogs were: breed 100.0%, age 98.9%, sex-neuter status 99.7%, insurance status 100.0% and adult bodyweight 66.9%.

Descriptive results were reported on 1462 Labrador Retrievers and 20,786 non-Labrador Retrievers (Table 1). The median age of Labrador Retrievers (5.23 years, IQR 2.13–8.97, range 0.17–17.7) was older than for non-Labrador Retrievers (4.35 years, IQR 2.13–5.23, range 0.01–20.46) (p < 0.001). The median adult bodyweight of Labrador Retrievers (32.00 kg, IQR 28.50–36.17, range 19.60–46.75) was heavier than for non-Labrador Retrievers (12.46 kg, IQR 7.88–22.60, range 1.41–85.00) (p < 0.001).

**Table 1:** Descriptive statistics for demographic characteristics in Labrador Retrievers (n = 1462) and non-Labrador Retrievers (n = 20,786) under primary veterinary care in the UK.

Variable	Category	Labrador Retriever count (%)	Non-Labrador Retriever count (%)	p value
Age (years)	≤ 3	489 (33.7)	7636 (37.1)	< 0.001
	3 to < 6	325 (22.4)	5218 (25.4)	
	6 to < 9	275 (19.0)	3731 (18.2)	
	9 to < 12	206 (14.2)	2393 (11.6)	
	≥ 12	155 (10.7)	1586 (7.7)	
Sex-neuter status	Male entire	455 (31.2)	6003 (29.0)	0.005
	Male neutered	374 (25.7)	4854 (23.4)	
	Female entire	321 (22.0)	5328 (25.7)	
	Female neutered	308 (21.1)	4538 (21.9)	
breed/sex mean bodyweight At/above or below	At or above	491 (33.6)	6337 (30.5)	0.005
	Below	539 (36.9)	7507 (36.1)	
	Not recorded	432 (29.5)	6942 (33.4)	
Insurance status	Insured	258 (17.6)	2718 (13.1)	0.001
	Not insured	1204 (82.4)	18,068 (86.9)	
Vet group	1	1 (0.1)	76 (0.4)	0.127
	2	451 (30.8)	6848 (32.9)	
	3	74 (5.1)	929 (4.5)	
	4	253 (17.3)	3554 (17.1)	
	5	683 (46.7)	9379 (45.1)	

The p value represents comparison of demographic variables between Labrador Retrievers and non-Labrador Retrievers.

Missing data were not included unless it accounted for a significant proportion of the variable (> 10%).

### Total disorder count in Labrador Retrievers compared with non-Labrador Retrievers

Of the Labrador Retrievers, 996/1462 (68.1%) were recorded with ≥ 1 disorder during 2016 compared with 13,667/20,786 (65.8%) of the non-Labrador Retrievers. After using multivariable methods to account for effects of age, sex-neuter status, at/above or below breed/sex mean bodyweight, insurance status and vet group, the odds of diagnosis with ≥ 1 disorder did not differ between Labrador Retrievers compared with non-Labrador Retrievers (odds ratio [OR] 1.00; 95% confidence interval [CI] 0.89–1.13; p = 0.967).

### Specific-level predispositions and protections

The 30 most common specific-level disorders in Labrador Retrievers and the 30 most common specific-level disorders in non-Labrador Retrievers resulted in a combined list of 35 specific-level disorders. The majority of these disorders overlapped, other than kennel cough, moist dermatitis, laceration, stiffness, coughing and papilloma which were exclusive to the 30 most common specific-level disorders in Labrador Retrievers. Conversely, heart murmur, flea infestation, atopic dermatitis, skin cyst, patellar luxation and retained deciduous tooth were exclusive to the 30 most common specific-level disorders in non-Labrador Retrievers.

**Figure 1:**
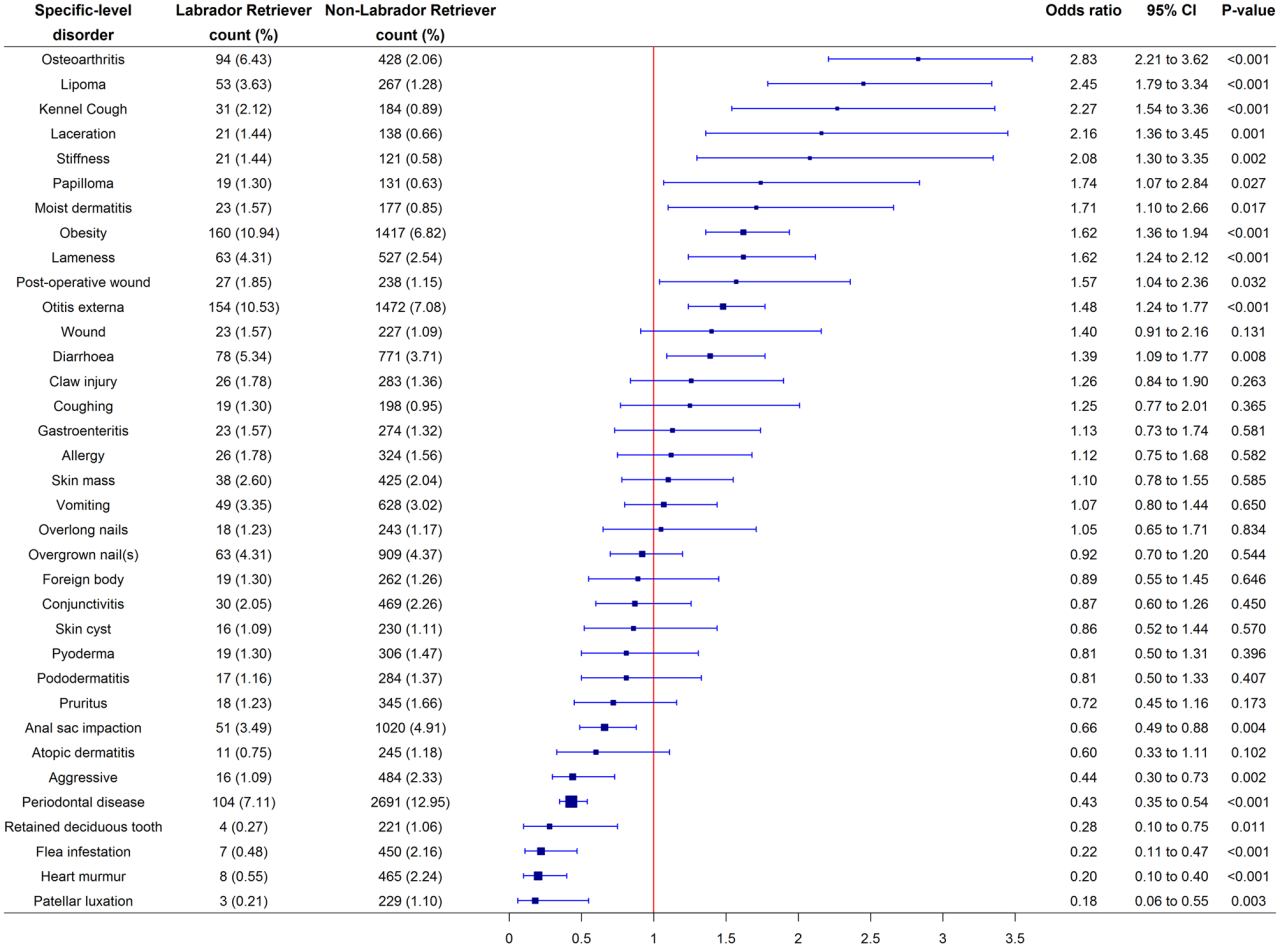
Forest plot of the prevalence and multivariable logistic regression odds ratios with corresponding 95% CIs (confidence intervals) for the combined list from the 30 most common disorders in Labrador Retrievers and the 30 most common disorders in non-Labrador Retrievers at a specific-level of diagnostic precision recorded in dogs under primary veterinary care at UK practices participating in the VetCompass Programme from January 1st 2016 to December 31st, 2016. Model variables accounted for included age, sex-neuter status, at/above or below breed/sex mean bodyweight, insurance status and vet group. Specific-level precision describes the original extracted terms at the maximal diagnostic precision recorded within the clinical notes.

After accounting for confounding using multivariable methods, Labrador Retrievers had significantly increased odds of 12/35 (34.3%) disorders compared to non-Labrador Retrievers. Disorders in Labrador Retrievers with the highest odds (i.e. predisposition) were: osteoarthritis (OR 2.83; 95% CI 2.21–3.62; p < 0.001), lipoma (OR 2.45; 95% CI 1.79–3.34; p < 0.001), kennel cough (OR 2.27; 95% CI 1.54–3.36; p < 0.001), laceration (OR 2.16; 95% CI 1.36–3.45; p = 0.001) and stiffness (OR 2.08; 95% CI 1.30–3.35; p = 0.002). Conversely, Labrador Retrievers had reduced odds (i.e. protection) for 7/35 (20.0%) specific-level disorders compared to non-Labrador Retrievers. The specific-level disorders with greatest protection were: patellar luxation (OR 0.18; 95% CI 0.06–0.55; p = 0.003), heart murmur (OR 0.20; 95% CI 0.10–0.40 (p < 0.001), flea infestation (OR 0.22; 95% CI 0.11–0.47; p < 0.001), retained deciduous tooth (OR 0.28; 95% CI 0.10–0.75; p = 0.011) and periodontal disease (OR 0.43; 95% CI 0.35–0.54; p < 0.001) (Fig. 1).

### Grouped-level predispositions and protections

The 30 most common grouped-level disorders in Labrador Retrievers and the 30 most common grouped-level disorders in non-Labrador Retrievers resulted in a combined list of 32 grouped-level disorders. The majority of these disorders overlapped, other than collapsed and oral cavity disorder which were exclusive to the 30 most common grouped-level disorders in Labrador Retrievers. Conversely, hernia and disorder not diagnosed were exclusive to the 30 most common grouped-level disorders in non-Labrador Retrievers.

**Figure 2:**
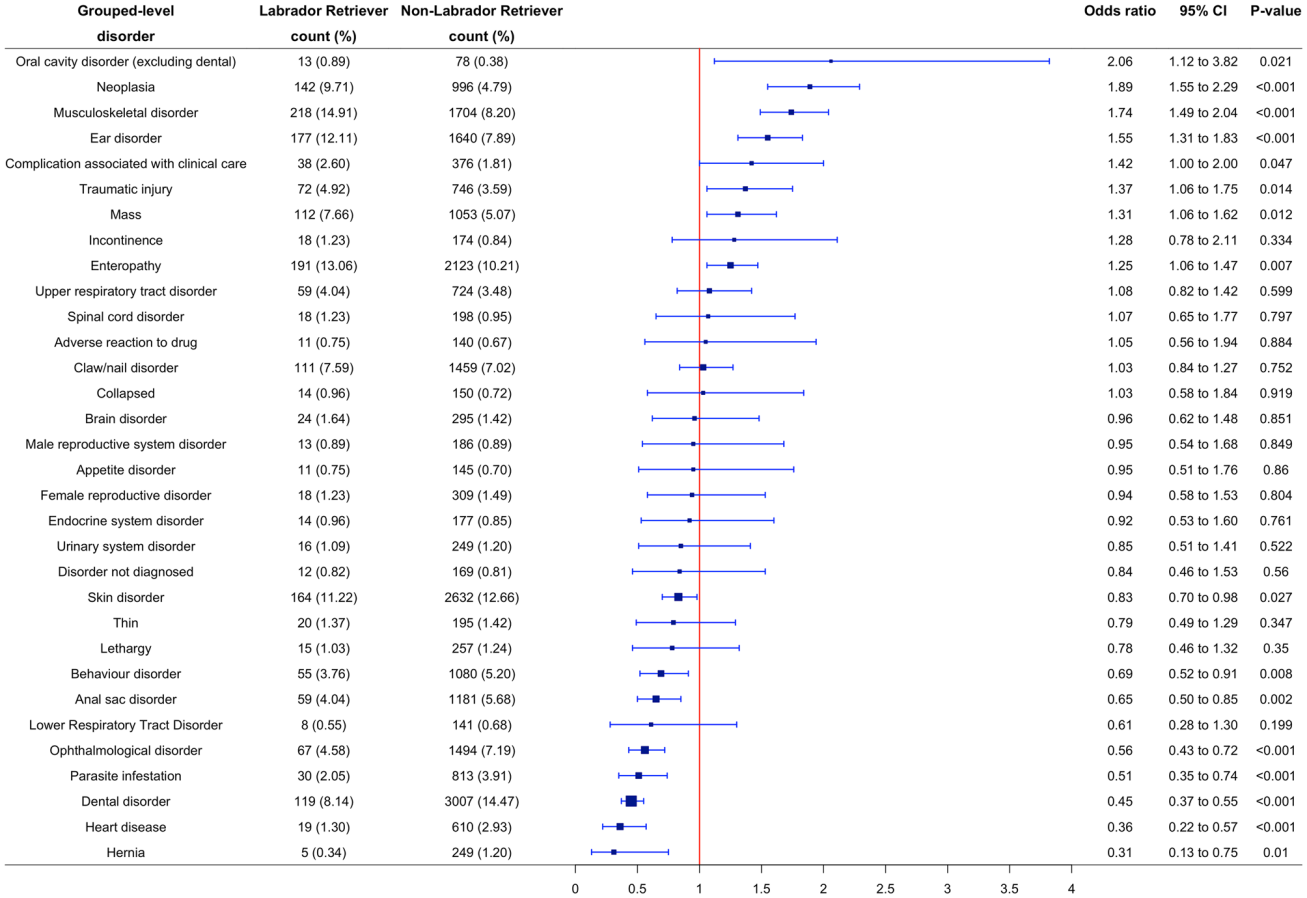
Forest plot of the prevalence and multivariable logistic regression odds ratios with corresponding 95% CIs (confidence intervals) for the combined list from the 30 most common disorders in Labrador Retrievers and the 30 most common disorders in non-Labrador Retrievers at a grouped-level of diagnostic precision recorded in dogs under primary veterinary care at UK practices participating in the VetCompass Programme from January 1st 2016 to December 31st, 2016. Model variables accounted for included age, sex-neuter status, at/above or below breed/sex mean bodyweight, insurance status and vet group. Grouped-level precision describes the original extracted terms mapped to a general level of diagnostic precision.

After accounting for confounding using multivariable methods, Labrador Retrievers had significantly increased odds of 8/32 (25%) disorders compared to non-Labrador Retrievers. The highest disorder predispositions were: oral cavity disorder (excluding dental) (OR 2.06; 95% CI 1.12–3.82; p = 0.021), neoplasia (OR 1.89; 95% CI 1.55–2.29; p < 0.001), musculoskeletal disorder (OR 1.74; 95% CI 1.49–2.04; p < 0.001), ear disorder (OR 1.55; 95% CI 1.31–1.83; p < 0.001) and complication associated with clinical care (OR 1.42; 95% CI 1.00–2.00; p = 0.047). Conversely, Labrador Retrievers had reduced odds of 8/32 (25%) grouped-level disorders compared to non-Labrador Retrievers. The grouped disorders with greatest protections were: hernia (OR 0.31; 95% CI 0.13–0.75; p = 0.010), heart disease (OR 0.36; 95% CI 0.22–0.57; p < 0.001), dental disorder (OR 0.45; 95% CI 0.37–0.55; p < 0.001), parasite infestation (OR 0.51; 95% CI 0.35–0.74; p < 0.001) and ophthalmological disorder (OR 0.56; 95% CI 0.43–0.72; p < 0.001) (Fig. 2). The Hosmer–Lemeshow test indicated no evidence of poor model fit (p > 0.05) in all multivariable models.

## Discussion

This is the largest study to date that reports the comparative risk across a range of common disorders between Labrador Retrievers and all remaining dogs under primary veterinary care. The study characterised the demography and health of a large cohort of 1462 Labrador Retrievers and 20,786 non-Labrador Retrievers under primary veterinary care in the UK. This current analytic study builds on a previous descriptive report of disorder prevalence in Labrador Retrievers under primary-veterinary care^27^ by using a larger, more recent and more comprehensive dataset that includes dogs of all breeds and types under primary veterinary care to compare disorder risk between Labrador Retrievers and all other dog types (including purebreds and crossbreeds). This has enabled reporting of relative predispositions to, and protections from, common disorders, using methods that have previously been restricted due to the inability to access the large data resources required for such analyses.

To date, much of the emphasis when reporting on breed health has focused on identifying disorders with increased risk^8^. However, reporting both disorder predispositions and protections offers a more balanced view of the overall health of the breed. The availability of large-scale data allows health comparison of individual breed against other groups across a range of disorders rather than just reporting risk for single disorders, which has been a limiting factor in many studies to date^28^. Disorder predispositions may have been more readily identified and reported in common breeds, due to the availability of adequate counts of these dogs in the general population. However, it is important that we understand about disorder protections also since it is possible that we may learn as much about how to breed healthier dogs by breeding towards low disorder risk as by breeding away from high disorder risk as is the current preference in dog breeding strategies^9^. In addition to information on predisposition and protections, we also need to consider absolute prevalence because even reduction from a high predisposition for a rare disorder may offer minimal overall health impact for a breed^46^. For example, the top predisposition in Labrador Retrievers at a grouped-level of diagnostic precision was oral cavity disorder (excluding dental) (OR 2.06), however this was a relatively rare disorder overall with the prevalence in Labrador Retrievers 0.89% compared with 0.38% in non-Labrador Retrievers.

At a specific level of diagnostic precision, Labrador Retrievers had higher odds of 12/35 (34.3%) disorders compared to non-Labrador Retrievers and had reduced odds of 7/35 (20.0%) disorders. At a grouped-level of diagnostic precision, Labrador Retrievers had higher odds of 8/32 (25.0%) disorders and had reduced odds of 8/32 (25.0%) disorders compared to non-Labrador Retrievers. A recent study with similar methodology based on Staffordshire Bull Terriers (SBTs), reported SBTs with higher odds of 4/36 (11.1%) specific-level disorders compared to non-SBTs and reduced odds of 5/36 (13.9%) disorders. At a grouped-level of diagnostic precision, SBTs had higher odds of 2/32 (6.3%) disorders and had reduced odds of 5/32 (15.6%) disorders compared to non-SBTs^43^. With significantly different risks between Labrador Retrievers and non-Labrador Retrievers for 19/35 (54.3%) specific-level disorders, these results show that the disorder profile of Labrador Retrievers is quite different to dogs that are not of this breed. However, with substantial counts of both predispositions (12) and protections (7) among these discordant disorders, and without having taken account of additional factors relating to each disorder such as duration and severity^47^, it is unclear whether the overall health of Labrador Retrievers can be concluded to be better or worse that non-Labrador Retrievers, but just different.

### Musculoskeletal disorders

Labrador Retrievers showed predisposition to musculoskeletal disorders at a grouped-level of diagnostic precision (OR 1.74), in agreement with previous reports that the Labrador Retriever is predisposed to several musculoskeletal conditions including hip and elbow dysplasia^36,48–50^, developmental orthopaedic diseases^51^, immune-mediated polyarthritis^52^, osteoarthritis^36,53^ and cranial cruciate ligament rupture^54^. The prevalence of musculoskeletal disorders reported in Labrador Retrievers in the current study (14.91%) lies between previously reported estimates from studies with similar designs based on Labrador Retrievers under primary-veterinary care (12.6–16.2%)^27,28^ and is similar to the prevalence of ‘extremity-related’ diseases reported in clinical data from Labrador Retrievers in the Netherlands (15.6%)^35^. At a specific-level of diagnostic precision, Labrador Retrievers had higher odds of osteoarthritis (OR 2.83) compared to non-Labrador Retrievers, slightly higher than a previous report using primary-veterinary care data (OR 2.47)^31^ and the highest overall predisposition identified in the current study. Labradors Retrievers also had increased odds of lameness (OR 1.62) and stiffness (OR 2.08), which are clinical signs commonly associated with musculoskeletal disorders^55^. However, clinical signs such as ‘lameness’ and ‘stiffness’ are not diagnostic and might be associated with a range of diagnoses, including osteoarthritis^56^. Therefore, the absolute risk of osteoarthritis in Labrador Retrievers may be higher than we report, which is concerning considering the reported negative impact of musculoskeletal disorders on canine welfare and lifespan^57^. The British Veterinary Association and KC’s hip and elbow dysplasia screening schemes have been running for over 50 years and over 20 years respectively, with the purpose of improving breeding programs to reduce the severity of breed predispositions to musculoskeletal disorders^58^. According to the current study, Labrador Retrievers are still at increased risk, which highlights that a review of the schemes and more stringent prevention methods may be appropriate and timely. However, the current study did not quantify any change in risk. It is estimated that approximately 30% of dogs are Kennel Club registered^59^, therefore it might be that the proportion of affected dogs differ between the Kennel Club and non-Kennel Club registered populations.

Despite the predisposition to overall musculoskeletal conditions reported in the current study, Labrador retrievers showed protection to patellar luxation at a specific-level of diagnostic precision (OR 0.18). This is in agreement with a previous study of KC-registered breeds which reported a significantly lower prevalence of patellar laxation in Labrador Retrievers compared to other breeds^36^. These recent studies contrast to earlier reports that suggested an overrepresentation of Labrador Retrievers diagnosed with patellar luxation^60–63^. Lateral patellar luxation has been observed more often in larger breed dogs^61^, however these earlier studies included relatively small numbers of dogs and used univariable analyses which might have led to confounding of the results^64^. As a breed exhibiting protection, Labrador Retrievers could be used as a model to explore conformational and other factors that may assist to develop strategies to reduce the prevalence in other higher-risk breeds.

### Obesity

Labrador Retrievers showed predisposition to obesity in the current study (OR 1.62), which is similar to odds ratios previously reported (OR 1.47–1.70)^29,45,65^. The welfare of dogs is compromised when they are overweight or obese, with obesity associated with a number of disorders including osteoarthritis, type II diabetes mellitus, lung problems and urinary and reproductive disorders^47,66^. The quality of life of affected dogs has been reported as improved after successful weight loss^67,68^. Amongst others previously stated, obesity has been co-morbidly associated with musculoskeletal disorders in dogs^29^. Predisposition to both musculoskeletal disorders and obesity in the Labrador Retriever suggests the probability of a causal link between obesity and musculoskeletal disorders, which gives further impetus to current moves to raise the status of obesity to be formally classified as a veterinary disease and to be prioritised within breeding and clinical programmes^69^. In consequence, it is important that practical preventative advice is given to owners about the susceptibility of Labrador Retrievers to obesity and associated musculoskeletal disorders to improve the welfare of the breed. It should be noted that evidence for obesity required information recorded within the EPR indicating that the dog was obese at any point during 2016. Given that bodyweight was missing for 29.5% of Labrador Retrievers and 33.4% non-Labrador Retrievers, it is possible that some of these dogs were obese but not recorded as such in the clinical notes. It is difficult to ascertain how this might vary by breed, but this finding could be used to focus efforts in encouraging veterinary professionals to routinely record bodyweight of all dogs. The focus of the current study was to report disorder predispositions and protections in Labrador Retrievers. However, given the predisposition identified and welfare impact, obesity and its comorbid conditions is an important area for future research.

### Neoplasia

Labrador Retrievers showed predisposition to neoplasia (OR 1.89) and mass disorders (OR 1.31) at a grouped-level of diagnostic precision, with estimated prevalences of 9.71% and 7.66% respectively. These values are similar to the respective estimates reported previously by studies of Labrador Retrievers with similar methodologies of 7.4–14.8% for neoplasia and 4.80–8.26% for mass disorders^27,28^. These results are supported by reported predispositions in Labrador Retrievers to neoplasms including lingual squamous cell carcinoma^70^, mast cell tumours^71–73^, soft tissue sarcoma^72^ melanomas^74^ and various neoplasms of the thoracic limb^75^.

Labrador Retrievers showed predisposition to lipoma (OR 2.45) at a specific-level of diagnostic precision in the current study. This odds ratio is comparable to that reported in a recent study, where Labrador Retrievers had the 3^rd^ highest odds of lipoma compared to crossbreeds (OR 2.19), behind the Dobermann Pinscher and Weimaraner^33^. These findings also support earlier research which identified breed predisposition to lipoma in Labrador Retrievers^32^. Whilst lipomas are often clinically unremarkable^33^, they ranked 4th among the top 8 disorders based on VetCompass Welfare Impact score, with a median annual duration of over 50% in affected dogs^47^. Therefore, considering the predisposition identified, high absolute prevalence value disorder duration, breed health reforms to reduce the prevalence of lipomas should be considered as worthwhile priorities. Labrador Retrievers showed predisposition to papilloma (OR 1.74) in the current study, which has not been previously reported from primary-care data. Prior studies based on primary-care data have reported prevalence of skin masses in Labrador Retrievers as 3.2% and 2.5% respectively^27,28^, although it is not possible to discern what proportion of these were papilloma. It should be noted that the number of Labrador Retrievers with papilloma in the current study was relatively small (n = 19), therefore this predisposition should be interpreted in line with low prevalence as being of lower priority for the breed. However, a recent study has highlighted the oncogenic potential of papillomas, with benign papillomas transforming in to malignant carcinomas^76^. Veterinarians should be aware of this oncogenic potential and discuss with owners of affected dogs.

### Respiratory disorders

Previous studies based on primary-care data have not reported the prevalence of kennel cough, but Labrador Retrievers were identified as predisposed (OR 2.27) in the current study, in contrast to a KC-registered breed based study which did not identify Labrador Retrievers with significantly higher or lower within breed prevalence of kennel cough compared to the prevalence across breeds overall^36^. Increased odds of coughing or upper respiratory tract disorders in Labrador Retrievers were not identified in the current study and the prevalence of these two conditions reported was similar to those reported previously by studies with similar methodologies^27,28^. Coughing is a pathognomonic presenting sign of the kennel cough syndrome that can involve a spectrum of infectious agents including viral, bacterial and *Mycoplasma*^77^. It would be useful to know which (if any) particular pathogens Labrador Retrievers are susceptible to that cause kennel cough, and if environmental factors such as increased contact with other dogs has an impact on their susceptibility. Labrador Retrievers are a sociable breed and may be kept as family companions, service dogs, guide dogs or working gundogs^13^. Transmission of pathogens associated with kennel cough is both by direct contact and indirectly by aerosolized droplets from coughing or sneezing dogs and by fomites^78^. Therefore, if Labrador Retrievers spend an increased amount of time in contact with other dogs and humans compared with different breeds, the risk of kennel cough transmission might be higher. Further research is required to elucidate these relationships.

### Dental disorders

Labrador Retrievers showed protection to dental disorders at a grouped-level of diagnostic precision (OR 0.45) and protection to periodontal disease at a specific-level of diagnostic precision (OR 0.43), with estimated prevalences of 8.14% and 7.11%, respectively. These values are higher than the respective estimates reported previously by studies with similar methodologies of 3.8–5.5% for dental disorders and 3.2–4.3% for periodontal disease^27,28^. Our prevalence estimate for periodontal disease is considerably lower than previous estimates based on prospective planned dental examinations in Labrador Retrievers (56.6%)^79^ and estimates in dogs generally (60.0–86.3%)^80,81^, suggesting the prevalence of periodontal disease is underreported in primary-care data, although different study populations may in part account for this difference. The current study also reported that Labrador Retrievers had protection (OR 0.28) to retained deciduous teeth, which is in agreement with previous research in which Labrador Retrievers did not develop as many cases of unerupted teeth as Boxers^82^. Although Labrador Retrievers had protection to dental disorders in the current study, they still have a relatively high prevalence of such conditions. Exploration of the reasons why Labrador Retrievers are protected in comparison to other breeds may enable these protective factors to be increased so that the breed prevalence decreases further, and that the health and welfare of other breeds might also be improved.

### Cardiovascular disorders

Labrador Retrievers showed protection to heart disease at a grouped-level of diagnostic precision (OR 0.36) and had protection to heart murmur at a specific-level of diagnostic precision (OR 0.20), with estimated prevalence of 1.30% and 0.55% respectively, which are lower than respective estimates reported previously by O’Neill et al. (2014) of 1.5% in both cases^28^. The results of the current study agree with research that reported Labrador Retrievers have reduced odds of degenerative mitral valve disease and heart murmur in comparison with other breeds^38^. Based on a survey of owners, Wiles et al.^36^ reported that Labrador Retrievers had significantly lower prevalence of mitral valve disease, heart murmur and irregular heartbeat in comparison with other KC-registered breeds. Conversely, Labrador Retrievers have been reported to have predispositions to supraventricular tachycardia^83^, atrioventricular block^84^, pericardial effusion^85^ and congenital heart disease^86^. However, these specific diagnoses are less common and were not identified in the current study population in sufficient numbers to be included in the analysis. It might be that the historical selection of Labrador Retrievers as a working gundog^13^ has inadvertently resulted in selection against cardiac disease over many decades. Therefore, there may be value in using Labrador Retrievers in cross breeding programmes to outcross other breeds affected with specific cardiac predispositions^7^.

### Parasitic disorders

Labrador Retrievers showed protection to parasitic disorders at a grouped-level of diagnostic precision (OR 0.51) and flea infestation at a specific-level of diagnostic precision (OR 0.22), with estimated prevalence of 2.05% and 0.48%, respectively. Results for flea infestation in Labrador Retrievers has not been previously reported in primary-care studies, but our estimated prevalence for parasite infestation overall is between estimates reported previously by VetCompass studies with similar methodologies (1.7–3.5%)^27,28^. The prevalence of flea infestation in our results was considerably lower than reported in veterinary-based surveys of UK dogs (6.82–14.40%)^87,88^. The higher prevalence reported by Abdullah et al. might be because their methodology required veterinarians to adhere to a strict protocol when collecting samples, but the authors suggested that the true prevalence might be higher still, due to the poor sensitivity of qPCR methods. Additionally, Bond et al. found that almost half of dog owners did not know that their dogs had flea infestations, which could lead to considerable underestimation of the true prevalence via veterinary reporting^87^. Furthermore, it is likely that many owners buy anti-parasitic treatments over the counter, so it is not clear how accurately veterinary clinical data represents the true prevalence of parasite infestation. It is unclear why Labrador Retrievers have protection to parasite infection, but it could be due to their characteristically short coat-type, which has been associated with reduced likelihood of tick infestation^89^ and flea infestation^90^ in dogs, perhaps due to easier removal of the parasite during grooming (by the dog or owner) or due to the increased difficulty for the parasite to attach and grip to the coat. Labrador Retrievers’ affinity to water might also help to remove external parasites, especially if they are often bathed or allowed to swim outdoors. However, we did not separate out different confounding factors in the current study, therefore future studies that explore demographic, environmental, management and ownership factors as separate entities may help to further evaluate this finding.

### Limitations

The limitations of this study are similar to those in previous VetCompass publications with similar methodologies, largely based on the nature of retrospective analysis of electronic patient record data^28,91^. Many disorders reported in the current study have environmental risk factors and some of these may be breed-specific, however it is not possible to assess these with primary-care data alone. Further prospective studies might evaluate the influence of non-genetic factors, such as owner and dog behaviours and lifestyle, including diet and exercise, on the development of disorders identified in the current study. The severity and duration of disorders are not reported here, which could provide further insights into the nature and ranking of breed predispositions^47^. Comparing the relative number of predispositions to protections does not necessarily reflect breed health without a measure of severity, however the study findings highlight the types of conditions Labrador Retrievers are predisposed to and protected from.

We classified dogs as either ‘Labrador Retriever’ or ‘non-Labrador Retriever’ and removed any dogs with their breed information missing. The high percentage (28.8%) of crossbreeds in our non-Labrador Retrievers might have caused some dilution of the phenotype. For example, Labradoodles are a crossbreed of Labrador Retriever and Poodle and although not recognised by the KC, are an increasingly popular breed in the UK^92^. It is difficult to ascertain from the data what proportion of the non-Labrador Retrievers had partial Labrador Retriever progeny and to what extent these dogs affected the results. However, inclusion of crossbreeds with partial Labrador Retriever progeny would result in an underestimation of the breed effects, rather than overestimation. Therefore, we can be more confident that a true association exists for the results identified as statistically significant in this study.

A random subset of all dogs were included in this study due to temporal constraints on data coding, which might have caused some of the less common disorders to be underpowered and resulted in artificially inflated or deflated odds ratios. Therefore, the count, prevalence, odds ratios and confidence intervals should be considered together when interpreting the results. As discussed previously, presenting signs where more than one was listed were reported as the first given, which might have affected the data due to linguistic biases. Novel methods of diagnosis coding based on natural language processing recently developed by VetCompass might provide a solution to these problems in the future^93^.

We treated missing values as a separate category within the analysis. Using this approach may allow for residual confounding if the missing category is not homogenous^94^, but provides a less biased estimate than excluding this data^95^. We use multiple comparisons in this study and adherence to a cut-off *p* *value* of less than 0.05 to infer significance can lead to a Type 1 error of accepting false positive results. Furthermore, the small number of cases in some disorders that we report might have led to a type 2 error of accepting false negative results. We recommend that readers do not rely on the *p* *values* of odds ratios but consider the confidence levels, prevalence percentages and other results to interpret our findings^96^. The results for each of the disorders assessed should be interpreted as exploratory rather than confirmatory.

## Conclusion

This study evaluated a range of disorders and should be considered exploratory rather than confirmatory. The individual results should be confirmed in future a priori studies to increase confidence in the findings. With significantly different risks between Labrador Retrievers and non-Labrador Retrievers for 19/35 (54.3%) specific-level disorders, these results show that the disorder profile of Labrador Retrievers is quite different to dogs that are not of this breed. Labrador Retrievers show predisposition to some common disorders including osteoarthritis and lipoma but show protection to some common disorders including patellar luxation and heart murmur. The findings can alert current Labrador Retriever owners about key issues to monitor in their dogs, which may lead to earlier veterinary visits, quicker diagnosis and thus better prognosis. Further work should aim to elucidate the reasons for the predispositions and protections to disorders in order to benefit not only Labrador Retrievers but the wider canine community.

## Methods

The study population included all available dogs under primary veterinary care at clinics participating in the VetCompass Programme during 2016. Dogs under veterinary care were defined as those with either (a) at least one electronic patient record (EPR) (VeNom diagnosis term, free-text clinical note, treatment or bodyweight) recorded during 2016 or (b) at least one EPR recorded during both 2015 and 2017. Including dogs that had received care at the clinic in 2015 and 2017 broadened the inclusivity of the dogs in the study by including dogs that were under the care of the practice in 2016, but who did not receive any direct treatment in this period. Therefore, this study population should be more representative of the wider dog population. VetCompass collates de-identified EPR data from primary-care veterinary practices in the UK for epidemiological research^44^. Data fields available to VetCompass researchers include a unique animal identifier along with species, breed, date of birth, sex, neuter status, insurance status, and bodyweight, and also clinical information from free-form text clinical notes, summary diagnosis terms^97^ and treatment with relevant dates.

A cohort study design was used to estimate the 1-year (2016) period prevalence of the most commonly diagnosed disorders in Labrador Retrievers and for all other dogs^98^. Sample size calculations *in* *Epi* *info* *(CDC)*^99^ estimated that, based on a UK dog population of 10 million^100^, approximately 834 Labrador Retrievers and 16,673 non-Labrador Retrievers would be needed to detect an odds ratio of ≥ 2.0 based on an estimated 2.5% of Labrador Retrievers having a specific disorder during the study period, assuming 80% power and 95% confidence with a 20:1 ratio of non-Labrador Retrievers to Labrador Retrievers^43,99^. Ethics approval was obtained from the RVC Ethics and Welfare Committee (reference number SR2018-1652).

Breed information entered by the participating practices was cleaned and mapped to a VetCompass breed list derived and extended from the VeNom Coding breed list^97^. Dogs that were recorded as Labrador Retriever were categorised as Labrador Retriever and dogs recorded with any other breed or crossbreed term were categorised as non-Labrador Retriever. Neuter status was defined by the final available EPR neuter value and was combined with sex to create four categories: female entire, female neutered, male entire and male neutered. Adult bodyweight for an individual dog was defined as the mean of all bodyweight (kg) values recorded for the dog after reaching 18 months old. Mean adult bodyweight was reported overall and broken down by sex for all breeds with adult bodyweight available for at least 100 dogs. Based on previous reports^43,101^, bodyweight was further categorized as “at or above the breed/sex mean”, “below the breed/sex mean” and “no recorded bodyweight”. Age was defined as the age (years) at December 31, 2016 and was categorised into five groups: ≤ 3.0, 3.0 to < 6.0, 6.0 to < 9.0, 9.0 to < 12.0 and ≥ 12.0. Each veterinary clinic was categorised into one of the five practice groups included in the study. The practice groups included in the current study were distributed throughout the UK and were assigned a code during analysis to ensure anonymity. Vet group was included as a fixed effect to adjust for clustering at the clinic level. Insurance status was categorised as insured or not insured as recorded by the final available EPR. Missing data were recorded as “Not recorded” and, in line with previous reports based on EPR data, included in the analysis if this category accounted for > 10% of the study variable^102^. A systematic review on the approaches to handling missing data reported that complete-case analysis may seriously bias study results^95^, therefore we chose to include missing data as a separate category in the analysis if it accounted for a significant proportion (> 10%) of the study variable to minimize bias. This approach allows dogs with missing data for one variable to still contribute to the overall analyses based on information that is complete for other variables.

The VetCompass database allows for random ordering of unique animal identification numbers from the overall population. This facilitated random selection of the clinical records of a sample of dogs of all types. The clinical records were reviewed in detail to extract the most definitive (i.e. specific) diagnoses recorded for all disorders that existed during 2016^28^. Elective (e.g. neutering) or prophylactic (e.g. vaccination) clinical events were not included as disorders. No distinction was made between pre-existing and incident disorder presentations. Disorders described within the clinical notes using presenting sign terms (e.g. ‘vomiting’ or 'vomiting and diarrhoea'), but without a formally recorded clinical diagnostic term, were included using the first sign listed (e.g. vomiting). Obesity in dogs is a disorder that might be described in EPRs using different synonyms^103^. In line with previous publications based on primary care data, evidence for obesity required information recorded within the EPR indicating that the dog showed evidence of overweight to any degree during 2016^45,103^. The extracted diagnosis terms were mapped to a dual hierarchy of diagnostic precision for analysis: specific-level precision and grouped-level precision as previously described^28^. Briefly, specific-level precision terms described the original extracted terms at the maximal diagnostic precision recorded within the clinical notes (e.g. *inflammatory* *bowel* *disease* would remain as *inflammatory* *bowel* *disease*). The researchers made no attempts to second-guess underlying disorders in cases with presenting signs (e.g. lameness) recorded in lieu of formal diagnoses. Grouped-level precision terms mapped the original diagnosis terms to a general level of diagnostic precision (e.g. *inflammatory* *bowel* *disease* would map to *gastro-intestinal*).

Following data checking for internal validity and cleaning in Excel (Microsoft Office Excel 2013, Microsoft Corp.), analyses were conducted using SPSS version 24.0 (IBM Corp). The sex-neuter status, age, mean adult bodyweight, at/above or below breed/sex mean bodyweight and insurance status for Labrador Retrievers and non-Labrador Retrievers under veterinary care during 2016 were described.

One-year period prevalence values were reported separately for Labrador Retrievers and non-Labrador Retrievers. One-year period prevalence described the proportion of dogs from the study population recorded as having a given condition during 2016. A combined list of disorders was generated that included the 30 most common disorders in Labrador Retrievers and the 30 most common disorders in non-Labrador Retrievers so that equal weighting was given to the common disorders in both groups. Multivariable modelling using binary logistic regression was used to report the odds of each of these disorders in Labrador Retrievers compared with non-Labrador Retrievers. Predisposition was defined as higher breed odds for a disorder after accounting for other demographic factors. Conversely, protection was defined as lower breed odds for a disorder after accounting for other demographic factors. A predisposition or protection was considered significant at the 5% level i.e. p < 0.05. For each specific-level and grouped disorder, a separate multivariable model was created that included a consistent list of variables based on information theory^104,105^. Breed was the a priori factor of interest and the models additionally included age (years), sex-neuter status, at/above or below breed/sex mean bodyweight, insurance status and vet group as potential confounders. Individual bodyweight serves as a proxy of breed, therefore we included “at/above or below breed/sex mean bodyweight” to account for within breed variation. Relaxation on financial constraints to presentation for veterinary care, diagnostic procedures and surgical management through insurance has been shown to increase diagnostic probability in many conditions^33^, therefore insurance was considered as a confounding variable in the current study.

Normality of continuous variables (i.e. age and bodyweight) was assessed graphically and using the Kolmogorov–Smirnov (K–S) test for normality^106^, with continuous variables ummarised using median, interquartile range (IQR) and range. Mann–Whitney, Chi-square or Fisher’s exact tests were used as appropriate for comparison of demographic data between cases and non-cases^107,108^. Model fit was assessed with the Hosmer–Lemeshow Test^109^. Statistical significance was set at the 5% level. All figures were created in R statistical software (R version 3.6.1) using the “forestplot” package^110^.

### Ethics approval

Ethics approval was granted by the RVC Ethics and Welfare Committee (reference number URN Ref SR2018-1652).

## Data Availability

The datasets generated during and/or analysed during the current study will be made available at the RVC Research Online repository: https://researchonline.rvc.ac.uk/id/eprint/12650/.

## Abbreviations

CI: Confidence interval
EPR: Electronic patient record
IQR: Interquartile range
KC: The Kennel Club
OR: Odds ratio
UCD VGL: University of California Davis Veterinary Genetics Laboratory
